# Enteritis caused by *Campylobacter jejuni *followed by acute motor axonal neuropathy: a case report

**DOI:** 10.1186/1752-1947-4-101

**Published:** 2010-03-31

**Authors:** Biljana Miljković-Selimović, Dragana Lavrnić, Olga Morić, Lai-King Ng, Lawrence Price, Ljubica Šuturkova, Branislava Kocic, Tatjana Babić, Ljiljana Ristić, Slobodan Apostolski

**Affiliations:** 1 Department of Microbiology and Immunology, School of Medicine, University of Niš, Bul Dr Z Ɖinđića, 81, 18000 Niš, Serbia; 2 Institute of Neurology, School of Medicine, University of Belgrade, Dr Subotića 8, 11000 Belgrade, Serbia; 3 Republic Institute of Public Health, Dr Subotića 5, 11000 Belgrade, Serbia; 4 National Laboratory for Enteric Pathogens, National Microbiology Laboratory, The Canadian Science Centre for Human and Animal Health, 1015 Arlington Street, Winnipeg, Manitoba, R3E 2R2, Canada; 5 School of Pharmacy, University of Skopje, Bul Krste Misirkov bb 91000 Skopje, Former Yugoslav Republic of Macedonia; 6 Institute of Public Health in Niš, Center for Microbiology, Bul Dr Z Ɖinđića, 50, 18000, Niš, Serbia

## Abstract

**Introduction:**

*Campylobacter *species represent the main cause of bacterial diarrhea in developed countries and one of the most frequent causes of enterocolitis in developing ones. In some patients, *Campylobacter jejuni *infection of the gastrointestinal tract has been observed as an antecedent illness of acute motor axonal neuropathy, a variant of Guillain-Barré syndrome.

**Case presentation:**

We present a case of acute motor axonal neuropathy following infection with *Campylobacter jejuni *subspecies *jejuni*, biotype II, heat stable serotype O:19. A 46-year-old Caucasian man developed acute motor neuropathy 10 days after mild intestinal infection. The proximal and distal muscle weakness of his upper and lower extremities was associated with serum antibodies to *Campylobacter jejuni *and antibodies to ganglioside GM1. The electromyographic signs of neuropathic muscle action potentials with almost normal nerve conduction velocities indicated axonal neuropathy. Our patient's clinical and electrophysiological features fulfilled criteria for the diagnosis of an acute motor axonal neuropathy, a subtype of Guillain-Barré syndrome.

**Conclusion:**

As this is the first case of acute motor axonal neuropathy following infection with *Campylobacter jejuni *subspecies *jejuni *reported from the Balkan area, the present findings indicate the need for systematic studies and further clinical, epidemiological and microbiological investigations on the prevalence of *Campylobacter jejuni *and its heat stable serotypes in the etiology of Guillain-Barré syndrome and other post-infectious sequelae.

## Introduction

*Campylobacter jejuni *has been recognized as one of the most common causes of bacterial diarrheal illness in humans [1]. After *C. jejuni *infection, severe post-infectious sequelae may occur, such as post-infectious acute motor axonal neuropathy (AMAN), a subtype of Guillain-Barré syndrome (GBS) [2].

The clinical entity 'GBS' can be divided into three forms: acute inflammatory demyelinating polyneuropathy (AIDP), which has as its main characteristic feature the macrophage-mediated demyelination of the peripheral nerves; axonal pattern, with axonal involvement, consisting of AMAN; and rare acute motor-sensory axonal neuropathy (AMSAN). AIDP and AMAN are differentiated by their clinical, electrophysiological and serological manifestations [2]. Miller Fisher syndrome (MFS), a variant of GBS, is characterized by ophthalmoplegia, ataxia and areflexia. Conditions related to MFS are Bickerstaff's brainstem encephalitis (BBE) (ataxia and impaired consciousness, with or without ophthalmoplegia, and areflexia or hyporeflexia); acute ophthalmoparesis (extraocular muscle paralysis arising in the absence of ataxia or areflexia); ataxic GBS (which may be ataxic GBS, sensory ataxic GBS and pure sensory GBS in which ataxia is the dominant clinical feature, in the absence of either limb or craniopharyngeal weakness); and pharyngeal-cervical-brachial (PCB) weakness (oropharyngeal, neck and shoulder weakness in the absence of significant limb weakness, with facial palsy, blepharoptosis, absence of sensory disturbance and preserved tendon jerk in the legs) [2]. Some authors consider PCB, GBS, MFS and BBE to be forms of a continuous spectrum [3].

Studies have shown that lipopolysaccharides extracted from *C. jejuni *associated with GBS mimic human gangliosides in their structure [2]. Moreover, patients with GBS triggered by *C. jejuni *infection usually have more severe disease than those triggered by other microorganisms [4].

Serotyping based on heat stable (HS) antigens [5] is the preferred method for the investigation of the clonality of bacterial strains related to post-infectious polyneuropathies. Some HS *C. jejuni *serotypes such as HS O:19 are usually associated with GBS. HS serotypes observed in other GBS patients include O:1; O:2; O:4; O:4 complex (4, 13, 16, 43, 50); O:5; O:10; O:16; O:23; O:37; O:41; O:44 [6], and O:35 and O:13/65 [7].

There is a lack of evidence on the prevalence of *C. jejuni *in many geographical areas including Serbia as a triggering organism in the etiology of GBS and other post-infectious sequelae. In addition, there is a lack of evidence on serotype distribution and GBS associated strains for some geographical areas.

We present a case of AMAN, a subtype of GBS, following infection with *C. jejuni *subspecies *jejuni*, biotype II O:19. According to our knowledge, this is the first report of AMAN associated with *C. jejuni *in the Balkan region.

## Case presentation

A 46-year-old Caucasian man, a physician from a town in central Serbia, had nausea, fever, and had suffered watery diarrhea that lasted for one day. The diarrhea was self-limiting and he was treated only with antipyretic drugs. Ten days later, he felt muscle weakness in both upper and lower extremities. Proximal weakness in his arms resulted in difficulty in lifting them above his shoulders, and in his legs in difficulty climbing stairs and rising from a low chair. During the following days weakness also appeared in his distal arm muscles, and gradually increased in his upper and lower leg muscles until our patient became severely disabled. Three days prior to admission, our patient complained of muscular pain in both his legs, low back and shoulders.

On admission, seven days after the first symptoms occurred, our patient's mental status was normal and cranial nerves were not affected. Both nerves innervating the proximal and distal muscle groups were affected in the upper and lower extremities resulting in symmetric proximal and distal muscle weakness which ranged between 3 and 4 on the Medical Research Council (MRC) grading scale. Deep tendon reflexes were normal, with the exception of both triceps brachii reflexes which were abolished. Pathological reflexes were also absent. There was no sensory nerve dysfunction and no sphincter disturbances (Quantitative Sensory Testing, Semmes-Weinstein monofilament, 128 Hz tuning fork).

Electrophysiological investigation was performed three weeks after the beginning of the first symptoms. Electromyography (EMG) revealed polyphasic neuropathic muscle action potentials with reduced interference pattern and compensatory motor unit potentials in the proximal and distal muscles of his upper and lower extremities. The amplitude of motor evoked potentials was slightly reduced for both peroneal and right mediana and for the ulnar nerve. Motor and sensory conduction velocities were normal.

The following laboratory examinations were normal: blood sedimentation rate, complete blood count, blood glucose, serum electrolytes, urea nitrogen, creatinine, serum protein content, cholesterol, triglycerides, and liver and muscle enzymes. The protein level in the cerebrospinal fluid (CSF) was measured seven days after the beginning of muscle weakness and was slightly elevated at 0.59 g/L. According to laboratory standards, a normal upper limit is 0.46 g/L. Cell count was normal with 3 mononuclear cells/mm^3^. A normal cell value is less than 10 cells/mm^3^.

In view of a probable diagnosis of AMAN, our patient received from the first day of hospitalization a course of intravenous immunoglobulin (IVIG) at the standard dose of 0.4 g/kg/day: a daily dose of 30 g was administrated over a period of five days (our patient's weight was 75 kg). IVIG resulted in an immediate dramatic improvement. From the 11th day after his admission, in view of the positive stool cultures for *C. jejuni *(see below), he received 1500 mg of oral erythromycin daily in order to eradicate antigenic stimuli. On his 24th day of hospitalization, our patient's neurological status improved with residual mild weakness of the foot dorsiflexor muscles and minimal weakness of the proximal and distal muscles of his arms. Three weeks later he had no muscle weakness and his tendon reflexes were brisk.

Because of the clinical signs and the history of a preceding gastrointestinal infection, a stool sample was sent to the Republic Institute of Public Health, Belgrade, for microbiological examination. For the detection of thermophilic campylobacters the Columbia agar base was supplemented with 5% sheep blood and antibiotics (cefoperazone 1.5 g/L, colistin 106 U, vancomycin 1 g/L, amphotericin B 0.2 g/L), (bioMérieux, Marcy l'Etoile, France). Culture was incubated at 42°C for 48 hours in a microaerophilic atmosphere (biomérieux). Fortunately, due to a delay in sampling, the primary culture was seen as positive for *Campylobacter*, and the subculture of solid plate yielded a confluent growth of colonies typical for *Campylobacter*, which were oxidase- and catalase-positive with a characteristic microscopic appearance, with S-shaped and curved rods. Identification, biotyping and serotyping were performed at the Bacteriology and Enteric Disease Program, National Microbiology Laboratory, Winnipeg, Manitoba, Canada. A strain presumptively identified as *Campylobacter *was differentiated to the species level by a combination of biotyping tests [8] and by use of a polymerase chain reaction (PCR)-based restriction fragment length polymorphism (RFLP) test [9]. Heat stable serotyping was performed using Penner's method [5]. The strain was identified as *C. jejuni *subspecies *jejuni *biotype II HS O:19, sensitive to erythromycin.

Antibodies to *C. jejuni *were determined by indirect immunofluorescence assay using as antigen methanol-fixed whole bacteria of the *C. jejuni*. Five pools, each consisting of 50 sera of healthy blood donors, were used as controls.

Autoantibodies to peripheral nerve gangliosides, including monosialoganglioside (GM1), disialoganglioside (GD1a) and asialoganglioside (ASGM1) were determined by enzyme-linked immunosorbent assay (ELISA), using methanol-fixed antigen-coated 96-well micro-ELISA plates and peroxidase-conjugated secondary anti-immunoglobulin G (IgG) and immunoglobulin M (IgM) goat antibodies.

On the day of the admission, serum antibodies to *C. jejuni *were positive in a titer of 1:128, while the control sera had titers of up to 1:8. On day 24, the antibody titers were still positive at 1:32. The IgG anti-GM1 antibodies were positive in the first serum sample (day 4) in a significant titer (1:16,000, with negative controls 1:400) and changed to negative (1:400) on days 10 and 19 after admission. There was no serum reactivity against gangliosides GD1a and ASGM1.

## Discussion

So far, there have been no reports about the incidence of such cases in Serbia. The neurological signs of a peripheral neuropathy, the history of a preceding gastrointestinal infection, the isolation of *C. jejuni *serotype O:19 from the patient's stool and the positive IgG anti-GM1 antibodies are typical of a *C. jejuni*-associated GBS. In the present case, electrophysiological findings indicated axonal neuropathy which was in accordance with antecedent *C. jejuni *infection and positive anti-GM1 antibodies, and in combination with neurological finding was compatible with the diagnosis of AMAN.

Although GBS is characterized by areflexia, particularly in patients with severe flaccid paralysis, in the present case reflexes were retained (apart from in the triceps). In patients with mild and moderate muscle weakness with preserved active movement deep tendon reflexes may be lost in some but not all muscle groups. Some occasional cases with unusually brisk deep tendon reflexes have been reported [10].

Serological finding of anti-campylobacter antibodies, supporting results obtained by cultivation, and the finding of anti-ganglioside antibodies completed the picture of possible cross-reactivity as the mechanism of axonal lesion. Indeed, the role of anti-GM1 antibodies in GBS has been extensively investigated [11]. An experimental animal model of GBS induced by inoculation of GM1 ganglioside confirms the role of anti-GM1 antibodies in the immunopathogenesis of GBS [12]. Patients with AMAN and negative anti-GM1 antibodies may have antibodies to peripheral nerve myelin glycoprotein cross-reactive with GM1 [13].

Although our patient recovered without complications, GBS is not a mild disease. Population-based studies show mortality of approximately 10%, and a requirement for artificial ventilation in approximately 25% of cases [14].

Concerning the bacteriological diagnosis of a *C. Jejuni*-associated GBS, a lag time of 1-3 weeks between campylobacter infection and the onset of GBS must be taken into account. In our particular case, AMAN occurred 10 days after the onset of the campylobacter infection. *C. jejuni *was recovered in the sample taken on day 31 after the onset of diarrheal disease. Further excretion of microorganisms was not examined. For the recovery of the strain, only selective solid medium was used. Bacteria were present in the small sample, and sub-cultivation produced a pure culture and characteristic growth.

Consistent reports on the prevalence and characterization of thermophilic campylobacter strains isolated from all over the world are yet to be combined globally for research purposes. The characterization of thermophilic campylobacter strains is not necessary for routine diagnostic procedures since the disease is often mild and self-limited, and free of complications. However, some characteristics of clinical presentations, such as chronic post-infectious sequelae, are related to certain HS serotypes which may vary by geographical area; and the epidemiology of campylobacter-associated GBS cannot be determined without the use of typing schemes.

## Conclusion

*Campylobacter jejuni *enterocolitis was diagnosed in our patient with the AMAN form of GBS, suggesting a close association between the bacterial infection and the severe acute neuropathy, supporting the general hypothesis of cross-reactivity between bacteria and human peripheral neural tissue. After the administration of therapy including an antibacterial drug, our patient recovered fully despite the general poor prognosis in a case of campylobacter post-infectious sequelae. Indeed, besides neurological examinations, specific microbiological and immunological examinations are needed for etiological GBS diagnosis. As this is the first case of AMAN following infection with *C. jejuni *subspecies *jejuni*, reported from Serbia, further clinical, epidemiological and microbiological investigations on the prevalence of *C. jejuni *and its HS serotypes in the etiology of GBS and other post-infectious sequelae are warranted.

## Consent

Written informed consent was obtained from the patient for publication of this case report and any accompanying images. A copy of the written consent is available for review by the Editor-in-Chief of this journal.

## Competing interests

The authors declare that they have no competing interests.

## Authors' contributions

BMS conceived the study, and co-ordinated and drafted the manuscript; DL provided information about the clinical course of the patient; OM, BK, TB, LR performed microbiogical investigation; LP performed PCR identification, biotyping and serotyping; LS performed the immunological assay; LKN and SA participated in its design and helped to draft the manuscript. All authors read and approved the final manuscript.
